# The Emergence of the Genus *Comamonas* as Important Opportunistic Pathogens

**DOI:** 10.3390/pathogens11091032

**Published:** 2022-09-12

**Authors:** Michael P. Ryan, Ludmila Sevjahova, Rachel Gorman, Sandra White

**Affiliations:** Department of Applied Sciences, Technological University of the Shannon Midwest, Moylish, V94 EC5T Limerick, Ireland

**Keywords:** *Comamonas*, nosocomial infection, environmental bacteria

## Abstract

*Comamonas* spp. are non-fermenting Gram-negative bacilli. They were first discovered in 1894, and since then, twenty-four species have been characterized. The natural habitat of these bacteria is soil, wastewater/sludge, fresh water such as ponds and rivers, and the animal intestinal microbiome. They were also isolated from industrial settings, such as activated sludge and polluted soil, and from the hospital environment and clinical samples, such as urine, pus, blood, feces, and kidney. *Comamonas* spp. are associated with environmental bioremediation and are considered an important environmental bacterium rather than a human pathogen. However, in the 1980s, they became a concern when several human infections associated with these species were reported. Here, the *Comamonas* genus was examined in terms of its members, identification techniques, and pathogenicity. Seventy-seven infection cases associated with these microorganisms that have been discussed in the literature were identified and investigated in this project. All relevant information regarding year of infection, country of origin, patient information such as age, sex, underlying medical conditions if any, type of infection caused by the *Comamonas* species, antibiotic susceptibility testing, treatment, and outcomes for the patient were extracted from case reports. The findings suggest that even though *Comamonas* spp. are thought of as being of low virulence, they have caused harmful health conditions in many healthy individuals and even death in patients with underlying conditions. Antimicrobial treatment of infections associated with these species, in general, was not very difficult; however, it can become an issue in the future because some strains are already resistant to different classes of antibiotics. Therefore, these pathogens should be considered of such importance that they should be included in the hospital screening programs.

## 1. Introduction

The growing range of severe infections caused by little-known non-fermenting Gram-negative rods is developing into a major cause of concern. These pathogens are opportunistic, infecting patients undertaking medical treatments in hospital and immunocompromised individuals outside of clinical locations. Bacterial species, including *Ralstonia* spp., *Ochrobactrum* spp., *Pseudomonas aeruginosa*, *Sphingomonas paucimobilis*, and *Brevundimonas* spp., all belong to this group [1,2,3,4,5,6]. Other emerging Gram-negative, non-fermenting rod bacteria that can cause potentially severe infections are members of the β-proteobacterial genus *Comamonas* [7].

*Comamonas* spp. have been isolated from a broad variety of environments, including water, aircraft water, soil, plants, and animals [8,9,10,11,12]. Several *Comamonas* spp. have been investigated for their potential to degrade xenobiotic pollutants and for heavy metal detoxification under a variety of environmental conditions [13,14,15,16,17,18,19]. *Comamonas* spp. are thought to be of low virulence. They have, however, caused infections, including serious infection such as septicemia or endocarditis, in immunocompetent hosts [20,21,22].

Analysis of the scientific/medical literature showed wide-ranging types of infections resulting from *Comamonas* spp. These were resistant to numerous different antibiotics. The data uncovered that this genus is a more commonplace pathogen than hitherto believed, with numerous infections/conditions caused by *Comamonas* spp. being severe and incapacitating. The purpose of this study was to give a general summation of infections caused by *Comamonas* spp., any underlying disorders/illnesses in patients that predispose them to infections with these bacteria and the antibiotic therapies that can be used for the management of these infections to aid medical professionals.

## 2. Genus *Comamonas*

**Figure 1:**
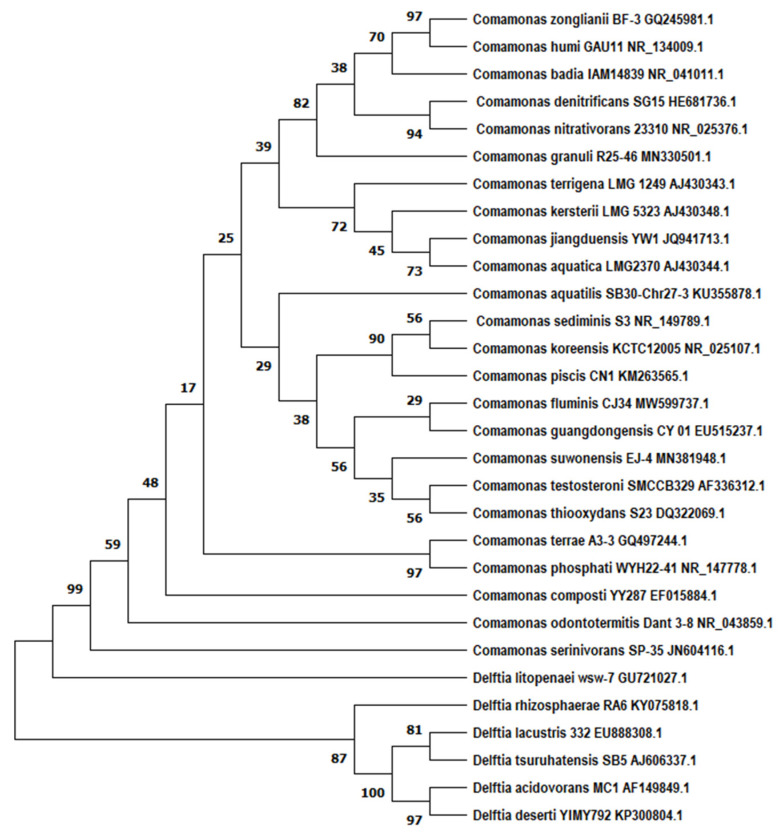
Phylogenetic tree of the genus *Comamonas* (accession numbers are given alongside species name) with the closely related genus *Delftia*. The tree was built with 16S rDNA genes (partial sequences of ~1400 bp) using neighbor-joining with the Tajma-Nei method utilizing the MEGA 11 software package. Bootstrap values are represented by numbers at nodes. These are based on 1000 resamplings. Bar, 0.0050 substitutions per site [26,27]. It should be remembered that these analyses are based upon 16S rDNA and, as such, are suggestive only.

Previously designated as *Pseudomonas* rRNA homology group III, the family Comamonadaceae now includes the genera *Comamonas*, *Delftia* and *Acidovorax*. The genus *Comamonas*, assigned to the Comamonadaceae lineage in the β-Proteobacteria, was originally proposed by Davis and Park [23] and the name validly published with the revival of the genus and the type species *Comamonas terrigena* by De Vos et al. [24]. In 1987, two *Pseudomonas* species, *Pseudomonas acidovorans* and *Pseudomonas testosterone*, were transferred to the genus *Comamonas* as *Comamonas acidovorans* and *Comamonas testosteroni*, respectively [24]. Based on a detailed 16S rRNA gene sequence-based phylogenetic study of the Comamonadaceae *C. acidovorans* was transferred as a type species to the novel genus *Delftia* as *Delftia acidovorans* [25]. Since then, the *Comamonas* genus has expanded to 24 species (see Table 1). The phylogenetic relationship between all *Comamonas* spp. described to date is presented in Figure 1.

## 3. Identification of *Comamonas* spp.

The *Comamonas* species are Gram-negative and comprised of straight or slightly curved rods or spirilla. They are usually 0.5 to 2 by 1 to 6 µm. They are generally motile by means of polar or bipolar tufts of 1–5 flagella (excepting *C. koreensis*). They are aerobic and chemoorganotrophic (De Vos et al., 2015) [50]. Some of the species are non-pigmented, some appear to be cream or yellow-white in color, and some can produce a brown halo around them (Willems and De Vos, 2006) [51], but they do not produce fluorescent pigments. Colonies appear pink-pigmented with a slimy and convex surface on blood agar. No hemolysis was observed on blood and chocolate agar. They are aerobic, oxidase and catalase-positive, non-spore formers, glucose non-fermenters, and chemoorganotrophic. Good growth was observed on media that contained peptone, organic acids, and amino acids (Public Health England, 2015) [52].

## 4. *Comamonas* spp. Virulence

*Comamonas* spp. are believed to be of low virulence. A study of the pangenome of 34 *Comamonas* genomes, however, showed that they have a diverse array of virulence factors, including polysaccharide biosynthesis for adherence and anti-phagocytosis, a motility system and metabolic enzymes for adaptation in vivo. All sequenced, clinically-isolated Comamonas strains and a number of environmental *Comamonas* spp. contain hemolysin genes. These analyses indicated that virulence might be species-specific as certain virulence factors are conserved in pathogenic-like strains [53]. 

## 5. *Comamonas* spp. Outbreaks

The overall knowledge gained from research into the scientific and medical literature can be seen in Table 2, Table 3 and Table 4. These tables show the year when the infection happened (if not available, the year of publication was used), country where the infection happened, patient information (age, sex, any reported underlying medical conditions), type of infection caused by the *Comamonas* infection, antimicrobial testing (susceptibility and resistance), treatment (focusing on the antibiotic therapies used) and patient outcome. 

Table 2, Table 3 and Table 4 illustrate 77 instances of infection caused by *Comamonas* spp. that were found in literature sources. It was found that only five *Comamonas* species (out of 24 species so far identified) have caused infections in humans. Most of these infections were caused by *Comamonas testosteroni* (50 instances—65.3%), other infections were due to *Comamonas kerstersii* (23 instances—29.8%), *Comamonas aquatica* (1 instance—1.3%), *Comamonas thiooxydans* (1 instance—1.3%), and *Comamonas terrigena* (1 instance—1.3%). In 47 instances (61%) out of 76, the patients had underlying conditions. Twenty different types of infection were caused by the different Comamonas species. These included pneumonia, polymicrobial bacteremia, bacteremia/septic shock, purulent meningitis, and sepsis.

Most patients had one underlying condition, seven had patients with two underlying conditions, and eight had patients with multiple underlying conditions (for example, obesity and diabetes). The most abundant of these underlying conditions were diabetes (in 8 patients—10.3%), various types of cancer (in 5 patients—6.5%) and alcoholism (in 4 patients—5.2%). Other major underlying conditions included obesity (in 3 patients—3.9%), hypertension (in 4 patients—10.9%), and renal failure (in 3 patients—3.9%). A full list of underlying conditions can be seen in Table 2, Table 3 and Table 4. A total of 70 patients (92.1%) were treated successfully and recovered fully, and 6 patients (7.8%) died. All patients who died due to *Comamonas* spp. infection suffered from one or more underlying conditions. These cases are discussed in more detail below. Surprisingly, to date, no pseudo-outbreaks have been found associated with *Comamonas* spp. 

Most of the reported infections caused by *Comamonas* spp. appear to be community-acquired [22].

### Death Associated with Comamonas spp. Infection

Six instances of death associated with *Comamonas* spp. infection have been reported. All six cases were linked to *C. testosterone* (Table 2). The first two instances were reported by Barbaro et al. [54]. In one of these instances, a mother who was an intravenous drug abuser gave birth to a premature baby, and this newborn baby died of sepsis caused by *C. testosteroni* infection 24 h after he was born. The second instance was very similar as it was also associated with sepsis due to *C. testosteroni* infection in a premature baby who was stillborn by an intravenous drug abuser mother. The third instance of death was reported in 2008 by Jin et al. [55]. In this case, a 54-year-old homeless man alcoholic was hit by a car, he received multiple fractures of the facial bones and was hospitalized. He was diagnosed with multiple cerebral and cerebellar infarcts, which resulted in changed mental status. He died 15 days after the injury. An autopsy revealed diffuse purulent meningitis due to *C. testosteroni* infection. In the fourth instance reported by Swain and Rout, a 50-year-old woman who suffered from diabetes and had a chronic renal disease was hospitalized for bacteremia and septic shock [56]. She was treated with piperacillin-tazobactam antibiotics until *C. testosteroni* was identified. The microorganism was found to be resistant to piperacillin–tazobactam, so treatment was then changed to cefoperazone–sulbactam. However, despite this, the woman died due to septic shock. The fifth instance of death associated with *Comamonas* spp. was reported in 2017 by Yasayancan and Koseoglu [57]. A 68-year-old man with lung cancer and adrenal metastasis was diagnosed with polymicrobial bacteremia due to *C. testosteroni*, *Staphylococcus haemolyticus*, and *Acinetobacter baumannii* infection. The patient died on the 16th day, despite suitable treatments against these pathogens. The last reported instance of death due to *C. testosteroni* infection was reported in 2018 by Cetin et al. A 10-year-old boy with serious underlying conditions (cerebral palsy, scoliosis, and long-term support with home mechanical ventilation) was diagnosed with pneumonia due to *C testosteroni* infection [58]. The patient was treated with appropriate antimicrobial therapy, and after 21 days of treatment infection was cured but due to the patient’s poor health conditions, he died on day 50 of hospitalization. No deaths have been associated with *C. kerstersii* or any other *Comamonas* spp (Table 3 and Table 4).

**Table 2:** Incidences of *Comamonas testosteroni* infection from 1987 to 2022. Main characteristics of the case reports.

Author (Ref.)	Year	Sex/Age	Country	Co-Morbidity	Type of Infection	Susceptible to *	Resistance to *	Antibiotic Treatment	Outcome
Atkinson et al. 1975 [59]	1966	F/31 yr old	USA	Rheumatic heart disease	Septicemia	N/A	N/A	Kanamycin, Tetracycline	Full recovery
Grover Smith, 1979 [60]	1979	M/48 yr old	USA	Atrophic right leg	Pyarthrosis Septicemia	Amikacin, Ampicillin, Carbenicillin, Cephalothin, Chloramphenicol, Colistin, Gentamicin, Kanamycin, Tetracycline, Tobramycin	N/A	Cephalothin, Gentamicin. Followed by Ampicillin for 21 days.	Full recovery
Barbaro et al., 1987 [54]	1983	M/31 yr old	USA	None	Perforated appendix	N/A	N/A	Cefoxitin then drainage, then Ampicillin, Clindamycin, Gentamicin	Full recovery
Barbaro et al., 1987 [54]	1983	M/11 yr old	USA	None	Perforated appendix	N/A	N/A	Ampicillin, Clindamycin, Tobramycin	Full recovery
Barbaro et al., 1987 [54]	1983	F/59 yr old	USA	Alcoholic	Cirrhosis	N/A	N/A	Cefoxitin	Full recovery
Barbaro et al., 1987 [54]	1983	F/24 yr old	USA	Iv drug abuse	Meningitis	N/A	N/A	Moxalactam, Nafcillin	Full recovery
Barbaro et al., 1987 [54]	1984	F/21 yr old	USA	Pregnant	Perforated appendicitis	Cefoxitin	N/A	Surgery, Iv Cefoxitin for 9 days	Full recovery
Barbaro et al., 1987 [54]	1984	F/12 yr old	USA	None	Perforated appendicitis	N/A	N/A	Cefoxitin	Full recovery
Barbaro et al., 1987 [54]	1985	F/84 yr old	USA	Congestive heart failure	Urine tract infection	N/A	N/A	Ampicillin	Full recovery
Barbaro et al., 1987 [54]	1985	M/24 yr old	USA	None	Perforated appendicitis	N/A	N/A	Cefoxitin	Full recovery
Barbaro et al., 1987 [54]	1985	F/New-born	USA	Maternal IV drug abuse, Premature birth	Sepsis	N/A	N/A	Ampicillin, amikacin	Died
Barbaro et al., 1987 [54]	1985	Stillborn	USA	Maternal IV drug abuse, premature birth	Sepsis	N/A	N/A	None	Died
Franzetti et al., [61]	1992	N/A	Italy	AIDS	Respiratory infection	N/A	N/A	Ceftazidime	Full recovery
Le Moal et al., 2001 [62]	2001	F/75 yr old	France	Breast cancer	Bacteremia	Aztreonam, Ceftazidime, Piperacillin, Ticarcillin	Amikacin, Ciprofloxacin, Fosfomycin	Ceftazidime, Gentamicin for 10 days	Full recovery
Arda et al., 2003 [63]	2003	M/50 yr old	Turkey	Undergone cholesteatoma operation	Purulent meningitis	Ceftriaxone, Ceftazidime, Meropenem	N/A	Ceftriaxone (were 3 mg/mL), Ceftazidime (0.75 mg/mL), and Meropenem (0.47 mg/mL), then changed to Meropenem, 3 g/day and operation to remove the cholesteatoma	Full recovery
Smith et al., 2003 [64]	2003	M/89 yr old	USA	N/A	Bacteremia	N/A	N/A	Levofloxacin	Full recovery
Cooper et al., 2005 [22]	2005	M/49 yr old	USA	None	Endocarditis	Ampicillin, Gentamicin, first, second, third generation Cephalosporins, Imipenem, Ciprofloxacin, Levofloxacin, Piperacillin, SXT, Tobramycin	N/A	Initially Cefipime, Gentamicin, switched to Ampicillin, then followed by surgery and 6 weeks of IV antibiotic treatment	Full recovery
Gul et al., 2007 [65]	2006	M/22 yr old	Turkey	None	Bacteremia due to perforated appendicitis	Ampicillin/Sulbactam, Amikacin, Cefazolin, Ceftazidime, Cefepime, Ciprofloxacin, Gentamicin, Imipenem, Levofloxacin, Piperacillin-Tazobactam, Imipenem, Meropenem, SXT, Tobramycin	N/A	Iv Cefazolin 1 g was given before surgery, Iv Cefazolin 1 g every 8 h after surgery	Full recovery
Abraham and Simon, 2007 [7]	2007	F/54 yr old	USA	Metastatic esophageal cancer, an indwelling central venous catheter	Bacteremia, septic shock	N/A	N/A	Cefepime, Vancomycin, Azithromycin, Drotrecogin alfa, Glucocorticosteroids, Norepinephrine Vasopressin, then was changed to Cefepime and Ciprofloxacin for 16 days	Full recovery
Garolo et al., 2007 [66]	2007	M/63 yr old	Poland	Lumbar discectomy	Spondylodiscitis	N/A	N/A	Eicoplanine (600 mg e.v./day), Ciprofloxacin (400 mg 2 times/day), then Ciprofloxacin, Cotrimoxazole	Full recovery
Jin et al., 2008 [55]	2008	M/54 yr old	USA	Alcoholic	Purulent Meningitis	N/A	N/A	Moxifloxacin	Died
Reddy et al., 2009 [67]	2009	F/82 yr old	India	Diabetes, Cataract surgery	Post-operative endophthalmitis	Ceftazidime, Chloramphenicol, Ciprofloxacin, Gatifloxacin, Moxifloxacin, Ofloxacin	Amikacin, Gentamicin, Tobramycin	Intraocular injection of 1 mg Vancomycin and 1 mg Ceftazidime, Ciprofloxacin (oral and topical), steroids (oral and topical) and Cycloplegics then intravitreal Ceftazidime (1 mg), topical ceftazidime	Full recovery
Katırcıoğlu et al., 2010 [68]	2010	M/83 yr old	Turkey	Hypertension and ischemic cerebrovascular incident	Sepsis	Amikacin, Ciprofloxacin, Piperacillin-Tazobactam	Aztreonam, Cefepime, Ceftriaxon, Ceftazidime, Cefoperazon-Sulbactam, Tobramycin, Imipenem	Piperacillin-Tazobactam, Amikacin for 10 days	Full recovery
Nseir et al., 2011 [69]	2011	F/64 yr old	Israel	Diabetes mellitus Patient on hemodialysis	Bacteremia (Catheter-related)	Ceftazidime, Gentamycin, Quinolones	Ampicillin Penicillin, Rocephin.	Vancomycin, ceftriaxone	Died
Ozden et al., 2011 [70]	2011	M/10 yr old	Turkey	Cerebral palsy, tracheostomy	Infection	N/A	N/A	Ceftriaxone, clarithromycin	Full recovery
Tsui et al., 2011 [71]	2011	M/73 yr old	Taiwan	Chronic hepatitis B, liver cirrhosis, hepatocellular carcinoma	Bacteremia	N/A	N/A	Radiofrequency ablation for liver tumor, Cefmetazon (1 g every 8 h), Gentamicin (60 mg every 8 h), then changed for IV Levofloxacin (500 mg once a day), oral Levofloxacin (500 mg every day) for 4 days	Full recovery
Tsui et al., 2011 [71]	2011	M/54 yr old	Taiwan	Alcoholic, Mild obstructive lung disease, replaced hip joints	Bacteremia	N/A	N/A	Iv Oxacillin (2 g every 6 h), Cephalosporin, then IV Ciprofloxacin (400 mg for every 12 h) 8 days	Full recovery
Farshad et al., 2012 [72]	2010	M/10 yr old	Iran	Brain Medullo-blastoma, chemotherapy	Bacteremia	Amikacin, Ampicillin, Aztreonam Ceftazidime, Ceftriaxone, Cefuroxime, Gentamicin, Cephalexin, Ciprofloxacin, Imipenem, Meropenem, Piperacillin/Tazobactam Tobramycin, Ticarcillin, Tetracycline,	N/A	Iv Ciprofloxacin (10 mg/kg/day for 21 days), Amikacin (15 mg/kg/day for 21 days)	Full recovery
Farshad et al., 2012 [72]	2010	F/19 yr old	Iran	Osteosarcoma, chemotherapy	Bacteremia, septic shock	Amikacin, Ampicillin, Aztreonam Ceftazidime, Ceftriaxone, Cefuroxime, Gentamicin, Cephalexin, Ciprofloxacin, Imipenem, Meropenem, Piperacillin/Tazobactam Tobramycin, Ticarcillin, Tetracycline	N/A	Iv Vancomycin (60 mg/kg/day for 14 days) and Imipenem (100 mg/kg/day for 14 days), then oral Ciprofloxacin (30 mg/kg/day for three weeks)	Full recovery
Al Ramahi et al., 2013 [73]	2013	M/47 yr old	Jordan	Renal failure, maintained on hemodialysis	Bacteremia	Cefepime, Ciprofloxacin, Cotrimoxzole, Levofloxacin, Ofloxacin, Polymyxin B, Tigecycline	Amikacin, Gentamicin, Imipenem, Meropenem, Piperacillin/Tazobactam with intermediate sensitivity for Ceftazidime	Cefepime (1 g daily for 14 days), then oral Cyclosporine 200 mg twice daily, Mycophenolate Mofetil 360 mg twice daily Prednisone 30 mg twice daily, oral INH 300 mg once daily	Full recovery
Bayhan et al., 2013 [74]	2013	M/16 yr old	Turkey	None	Peritonitis due to perforated appendicitis	Amicasin, Ampicillin, Ampicillin-Sulbactam, Ceftazidime, Cefazolin, Ciprofloxacin, Gentamicin, Imipenem, Piperacillin	Ceftriaxone, Cefuroxime, SXT	Removal of appendix, Saline peritoneal lavage, IV Amicasin, Ampicillin, Clindamycin (5 days)	Full recovery
Altun et al., 2013 [75]	2013	F/29 yr old	Turkey	End-stage renal failure, hypertensive nephrosclerosis, CAPD	Peritonitis	N/A	N/A	Iv Vancomycin, oral Ciprofloxacin (14 days)	Full recovery
Orsini et al., 2014 [76]	2014	F/80 yr old	USA	Hypertension, diabetes mellitus, hiatal hernia, osteoarthritis, cholelithiasis, obesity	Polymicrobial bacteremia	Ceftazidime, Carbapenems, Piperacillin/Tazobactam, SXT	N/A	Initially Ceftriaxone (2 g IV daily), then Nafcillin (2 g IV every 4 h), Cefazolin (1 g IV every 8 h) and Doripenem (250 mg IV every 8 h)	Full recovery
Swain and Rout, 2015 [56]	2015	F/50 yr old	India	Diabetes mellitus complicated with chronic renal disease	Bacteremia, septic shock	Ceftazidime, Cefoperazone-Sulbactam, Meropenem	Amikacin, Cefepime, Ciprofloxacin, Gentamicin, Piperacillin-Tazobactam	Piperacillin-Tazobactum (3.375 gm IV 6 hourly), then changed for Cefoperazone-Sulbactam	Died
Duran et al., 2015 [77]	2015	M/51 yr old	Turkey	Tachycardia	Endocarditis	Amikacin, Ciprofloxacin, Ceftazidime, Cefoperazone-Sulbactam, Cefepime, Colistin Tigecycline	Gentamicin, Imipenem, Meropenem, Netilmicin, Piperacillin-Tazobactam	Cardiovascular surgery, Ciprofloxacin	Full recovery
Kim et al., 2015 [21]	2015	F/42 yr old	Korea	Meningioma was removed 6 days before infection	Septic shock	N/A	N/A	Initially Piperacillin/Tazobactam, Levofloxacin, Metronidazole iv, renal replacement therapy, Immunoglobulin IV Meropenem/Levofloxacin, then ceftazidime with levofloxacin	Full recovery
Khalki et al., 2016 [78]	2015	N/A/18	Morocco	None	Acute appendicitis	Amoxicillin—clavulanic acid, Cefoxitin, 2nd and 3rd generation Cephalosporins, Gentamycin, Amikacin, Carbapenems, Ticarcillin, Piperacillin	Amino-penicillins, Aztreonam, Ciprofloxacin, Nalidixic acid, Norfloxacin, SXT	Surgery, Amoxicillin-clavulanic acid IV for 48 h, then taken orally for 8 days	Full recovery
Pekintürk and Akgüneş, 2016 [79]	2016	M/62 yr old	Turkey	Left hemiparesis and type II diabetes	Bacteremia	Amikacin, Ceftazidime, Cefepime, Ciprofloxacin, Gentamicin, Imipenem, Levofloxacin, Meropenem, Netilmicin, Piperacillin, Piperacillin-Tazobactam, Tetracycline Tigecycline, Tobramycin, SXT	Aztreonam, Colistin	N/A	Died
Parolin et al., 2016 [80]	2016	F/4 yr old	Italy	End-stage renal disease, idiopathic epilepsy	Peritonitis	N/A	N/A	Initially IV Ceftazidime, Teicoplanin, then changed to Ciprofloxacin for 3 weeks	Full recovery
Hung et al., 2017 [81]	2017	F/63 yr old	Taiwan	Hemodialysis patient	Acute Appendicitis	Ceftriaxone, Ceftazidime, Gentamicin	Ciprofloxacin	Cefazolin Followed by ceftriaxone	Full recovery
Ruziaki and Hashami, 2017 [82]	2017	F/1 yr old	Oman	None	Sepsis	Ceftriaxone, Ceftazidime, Cefipime, Ciprofloxacin, Gentamicin	N/A	Iv Ceftriaxone (80 mg per kg per dose once a day for 14 days	Full recovery
Yasayancan and Koseoglu, 2017 [57]	2017	M/68 yr old	Turkey	Lung cancer, adrenal metastasis	Polymicrobial Bacteremia	Cefepime, Colistin, Levofloxacin, Tigecycline	Gentamycin, Imipenem, Meropenem, Piperacillin–Tazobactam	Piperacillin–Tazobactam and ciprofloxacin iv, then Cefepime Teicoplanin, then Tigecycline/Colistin	Died
Tartar and Tartar, 2020 [83]	2017	M/14 yr old	Turkey	None	Perforated appendicitis	Amikacin, Ampicillin–Sulbactam, Ceftazidime, Cefazolin, Ciprofloxacin, Gentamicin, Imipenem, Piperacillin, SXT	N/A	Surgery, IV Cefazolin (100 mg/kg), Amikacin (15 mg/kg), Metronidazole (30 mg/kg).	Full recovery
Tartar and Tartar, 2020 [83]	2017	F/5 yr old	Turkey	None	Acute appendicitis	Amikacin, Ertapenem, Ciprofloxacin, Gentamicin, Imipenem, Piperacillin, SXT	Ampicillin–Sulbactam, Ceftazidime, Cefuroxime	Surgery, IV Cefazolin (100 mg/kg), Amikacin (15 mg/kg), Metronidazole (30 mg/kg)	Full recovery
Farooq et al., 2017 [20]	2017	F/65 yr old	India	Colostomy	Gastroenteritis	Amikacin, Cefepime, Cefoperazone/Salbactam, Ceftazidime, Colistin, Gentamicin, Imipenem Cotrimoxazole, Minocycline, Meropenem, Piperacillin/Tazobactam, Tigecycline	Aztreonam, Ciprofloxacin, Levofloxacin	Oral Ciprofloxacin (500 mg for 3 days), probiotics	Full recovery
Cetin et al., 2018 [57]	2018	M/10 yr old	Turkey	Cerebral palsy, scoliosis, supported with long-term home mechanical ventilation	Pneumonia	Amikacin, Ceftazidime, Cefepime, Imipenem, Levofloxacin, Meropenem, Netilmicin, Piperacillin, Piperacillin-Tazobactam, Tigecycline, SXT	Aztreonam, Ciprofloxacin, Colistin, Gentamicin, Tetracycline	Amikacin (1 × 225 mg), Piperacillin-Tazobactam (3 × 1.5 g) Vancomycin (4 × 150 mg),	Died
Lovell and Forde, 2019 [84]	2019	M/39 yr old	Barbados	Alcoholism, asthma, pancreatitis	Bacteremia	Cefepime, Cefotaxime, Ceftriaxone, Ciprofloxacin, Levofloxacin, Meropenem, Piperacillin-Tazobactam, SXT	Cefazolin, Ertapenem, Gentamicin	Initially Meropenem 1 g IV every 8 h, Fluconazole 800 mg IV, a 21-day course of Meropenem and a 14-day course of Fluconazole (unsuccessfully), then SXT	Full recovery
Tiwari and Nanda, 2019 [85]	2019	F/46 yr old	India	None	Bacteremia	Amikacin, Cefuroxime, Ciprofloxacin, Colistin Gentamicin, Imipenem, Meropenem, Tigecycline, Cotrimoxazole	Piperacillin-Tazobactam	Initially Piperacillin-Tazobactam, Vancomycin, then changed for Gentamicin (4 mg/kg/daily) and Imipenem (25 mg/kg 8 hourly) for 10 days	Full recovery
Buyukberber et al., 2021 [86]	2020	F/4yr old	Turkey	Previous urinary surgery	Urinary tract infection	Ceftazidime, Ciprofloxacin; Meropenem Piperacillin/tazobactam	Amikacin, Gentamicin, Imipenem, SXT	Amikacin Followed by Ceftazidime	Full recovery
Miloudi et al., 2021 [87]	2020	N/A/12	Morocco	None	Acute appendicitis	Aminoglycosides, Amoxicillin/Clavulanic acid, 2nd, and 3rd generation Cephalosporins, Carbapenems, Colistin, Ticarcillin	Ciprofloxacin, Norfloxacin, SXT	Appendectomy and surgical drainage, Amoxicillin/Clavulanic acid (3 g/24 h for 15 days)	Full recovery
Ayhancı et al., 2021 [88]	2021	M/51 yr old	Turkey	None	Bacteriemia	Amikacin, Ciprofloxacin Gentamicin, Levofloxacin, Imipenem, Meropenem	N/A	Levofloxacin 500 mg/day w	Full recovery
Sammoni et al., 2022 [89]	2022	M/16 yr old	Syria	Burn victim	Sepsis	Colistin	N/A	Cefazolin and Ceftriaxone Followed by Colistin-amikacin for 14 days	Full recovery

F—Female, M—Male, N/A—Not Available, SXT sulfamethoxazole-Trimethoprim. * Antibiotic susceptibility testing was carried out using a variety of methods, including disk diffusion testing, agar and broth dilution testing and E-testing methods.

**Table 3:** Incidences of *Comamonas kerstersii* infection from 2013 to 2022. Main characteristics of the case reports.

Author (Ref.)	Year	Sex/Age	Country	Co-Morbidity	Type of Infection	Susceptible to *	Resistance to *	Antibiotic Treatment	Outcome
Almuzara et al., 2013 [90]	2013	F/43 yr old	Argentina	Ovarian tumor with peritoneal metastases	Sigmoid perforation by foreign body (biliary stent), rectovaginal fistula, and colostomy	Amikacin, Ampicillin, Ampicillin-Sulbactam, Cephalothin, Cefoxitin, Cefotaxime, Ceftazidime, Cefepime, Colistin, Gentamicin, Imipenem, Meropenem, Piperacillin-Tazobactam, SXT	Ciprofloxacin	Ampicillin-Sulbactam, Piperacillin-Tazobactam, Ertapenem	Full recovery
Almuzara et al., 2013 [90]	2013	M/48 yr old	Argentina	None	Perforated appendix	Amikacin, Ampicillin, Ampicillin-Sulbactam, Cephalothin, Cefoxitin, Cefotaxime, Ceftazidime, Cefepime, Ciprofloxacin, Colistin, Gentamicin, Imipenem, Meropenem, Piperacillin-Tazobactam, SXT	N/A	Ampicillin-Sulbactam, Ciprofloxacin, Amoxicillin-Clavulanic acid	Full recovery
Almuzara et al., 2013 [90]	2013	F/10 yr old	Argentina	None	Perforated gangrenous appendix	Amikacin, Ampicillin, Ampicillin-Sulbactam, Cephalothin, Cefoxitin, Cefotaxime, Ceftazidime, Cefepime, Colistin, Gentamicin, Imipenem, Meropenem, Piperacillin-Tazobactam, SXT, Ciprofloxacin, Colistin, SXT	Ciprofloxacin	Ampicillin, Metronidazole, Gentamicin, and then Amoxicillin-Clavulanic acid	Full recovery
Almuzara et al., 2013 [90]	2013	F/21 yr old	Argentina	None	Perforated gangrenous appendix	Amikacin, Ampicillin, Ampicillin-Sulbactam, Cephalothin, Cefoxitin, Cefotaxime, Ceftazidime, Cefepime, Colistin, Gentamicin, Imipenem, Meropenem, Piperacillin-Tazobactam, SXT	Ciprofloxacin	Ampicillin, Metronidazole, Gentamicin	Full recovery
Biswas et al., 2014 [91]	2014	M/10 yr old	United Kingdom	None	Perforated appendix	Amikacin, Ceftazidime, Ciprofloxacin, Colistin, Gentamicin, Meropenem, Piperacillin-Tazobactam	N/A	Open appendectomy, Piperacillin-Tazobactam (5 days), Amoxicillin-Clavulanic acid, Ciprofloxacin	Full recovery
Biswas et al., 2014 [91]	2014	M/9 yr old	United Kingdom	None	Septic shock (due to perforated appendix)	Amoxicillin-clavulanic acid, Ceftazidime, Colistin, Gentamicin, Meropenem, Piperacillin-Tazobactam	Ciprofloxacin	Surgery, Amoxicillin-Clavulanic acid, Gentamicin, Metronidazole (intravenously, 3 days), Amoxicillin-Clavulanic acid (orally)	Full recovery
Opota et al., 2014 [92]	2014	M/65 yr old	Switzerland	Diabetes	Bacteremia with sign of diverticulosis	Ceftazidime, Ciprofloxacin, Meropenem, Imipenem, Minocycline, Levofloxacin, SXT	N/A	Imipenem-Cilastatin (10 days)	Full recovery
Almuzara et al., 2017 [93]	2017	F/54 yr old	Argentina	Obesity, hypertension, diabetes	Septic shock	SXT, Metronidazole	Piperacillin/Tazobactam, Vancomycin	SXT 15 mg/kg (intravenously every 12 h) and Metronidazole 500 mg (intravenously every 8 h), 30 days	Full recovery
Almuzara et al., 2017 [93]	2017	F/15 yr old	Argentina	None	Pelvic peritonitis due to genital tract infection	N/A	N/A	Ceftriaxone (intravenously 2 g/day, 6 days), Metronidazole (orally 500 mg/12 h, 8 days), Doxycycline (orally 100 mg/12 h, 8 days), Amoxicillin/Clavulanic acid (orally 500 mg/8 h, 14 days)	Full recovery
Almuzara et al., 2018 [94]	2018	F/5 yr old	Argentina	None	Urinary tract infection	Amikacin, Ampicillin, Ampicillin/Sulbactam, Cephalothin, Colistin, Cefotaxime, Ceftazidime, Cefepime, Ciprofloxacin, Gentamycin, Imipenem, Meropenem, Piperacillin-Tazobactam, SXT	Ceftriaxone	Piperacillin/Tazobactam (intravenously 200 mg/kg per day, every 8 h, 10-days), Amoxicillin/Clavulanic (orally 50 mg/kg per day, 14 days)	Full recovery
Zhou et al., 2018 [95]	2018	M/31 yr old	China	None	Acute peritonitis, perforated appendix (with abdominal abscess)	All except Ciprofloxacin Levofloxacin, SXT	Ciprofloxacin Levofloxacin, SXT	Exploratory laparotomy, appendectomy, tube drainage, Cefuroxime and metronidazole (14 days)	Full recovery
Liu et al., 2020 [96]	2020	M/62 yr old	China	None	Intra-abdominal infection due to perforated colon	Amikacin, Ceftazidime, Cefepime, Ciprofloxacin, Colistin Imipenem, Levofloxacin, Meropenem, Minocycline, Piperacillin-Tazobactam, SXT	Cephalothin, Cefotaxime, Ciprofloxacin, Gentamicin	Surgery (left thoracotomy exploration, repair of oesophageal hiatal hernia, laparotomy exploration, partial colectomy, colostomy), Piperacillin-Tazobactam (Intravenously 4.5 g, every 8 h, 14 days)	Full recovery
Palacio et al., 2020 [97]	2020	M/16 yr old	Uruguay	None	Acute appendicitis	Amikacin, Ampicillin Sulbactam, Ceftazidime, Cefepime, Gentamicin, Piperacillin/Tazobactam, Meropenem, Imipenem, Cotrimoxazole	N/A	Laparoscopic surgery, Piperacillin/Tazobactam (intravenously, 4.5 g every 6 h, 10 days)	Full recovery
Farfán-Cano et al., 2020 [98]	2020	M/14 yr old	Ecuador	None	Perforated appendicitis	N/A	N/A	Piperacillin/Tazobactam (14 days)	Full recovery
Farfán-Cano et al., 2021 [99]	2020	F/27 yr old	Ecuador	Obesity and being on lactation period	Acute appendicitis	N/A	N/A	Ciprofloxacin and Metronidazole IV for 10 days	Full recovery
Farfán-Cano et al., 2021 [99]	2020	M/29 yr old	Ecuador	None	Acute appendicitis	N/A	N/A	Conventional Appendectomy, Ciprofloxacin, and Metronidazole	Full recovery
Farfán-Cano et al., 2021 [99]	2020	M/68 yr old	Ecuador	None	Acute appendicitis	N/A	N/A	Laparoscopic appendectomy	Full recovery
Farfán-Cano et al., 2021 [99]	2020	F/16 yr old	Ecuador	None	Acute appendicitis	N/A	N/A	Conventional appendectomy, Ampicillin/Sulbactam + Metronidazole	Full recovery
Farfán-Cano et al., 2021 [99]	2020	F/16 yr old	Ecuador	Psoriasis	Acute appendicitis	N/A	N/A	Conventional appendectomy, Ampicillin/ Sulbactam	Full recovery
Rong et al., 2022 [100]	2022	M/82 yr old	Canada	Type 2 diabetes	Bacteremia	Ceftazidime, Gentamicin, Imipenem, Meropenem, Piperacillin/tazobactam, Tobramycin	Ciprofloxacin	Ppiperacillin-tazobactam Followed by intravenous Ceftriaxone (1 g/day)	Full recovery
Bennani et al., 2022 [101]	2002	M/8 yr old	Morocco	None	Acute appendicitis	N/A	N/A	Intravenous Amoxicillin-clavulanic acid, Gentamicin, and Metronidazole Followed by oral Amoxicillin-Clavulanic acid.	Full recovery

F—Female, M—Male, N/A—Not Available, SXT sulfamethoxazole-trimethoprim. * Antibiotic susceptibility testing was carried out using a variety of methods, including disk diffusion testing, agar and broth dilution testing and E-testing methods.

**Table 4:** Incidences of *Comamonas* spp. infection from 2000 to 2022. Main characteristics of the case reports.

Author (Ref.)	Year	Sex/Age	Country	Co-Morbidity	Type of Infection	Susceptible to *	Resistance to *	Antibiotic Treatment	Outcome
Sonnenwirth, 1970 [102]	1970	F/71 yr old	USA	Rheumatic heart disease	Endocarditis	Chloramphenicol, Oxytetracycline Tetracycline	Ampicillin, Cephalothin, Colistin, Penicillin, Streptomycin	Penicillin	Full recovery
Isotalo et al., 2000 [103] *Comamonas* spp.	2000	M/35 yr old	Canada	None	Tenosynovitis (From an animal bite)	N/A	N/A	Intravenous (IV) cefazolin at 1 g/8 h and gentamicin 80 mg/8 h for a total of 72 h	Full recovery
Kaeuffer et al., 2018 [104] *Comamonas aquatica*	2017	M/66 yr old	France	Diabetes, ischemic heart disease, removed sigmoid polyps	Bacteremia and septic shock	Amoxicillin-Clavulanic acid, Ceftazidime, Cefepime, Ciprofloxacin, Imipenem, Piperacillin-Tazobactam	N/A	Norepinephrine, Cefotaxime, Ciprofloxacin (10 days)	Full recovery
Guo et al., 2021 [105] *Comamonas thiooxydans*	2021	F/60 yr old	China	Kidney stones.	Urinary Tract Infection	Chloramphenicol, Imipenem, SXT	Amikacin, Aztreonam, Ceftazidime, Cefepime Ciprofloxacin, Gentamicin, Levofloxacin	Imipenem-cilastatin 1 g IV for 1 month to fight	Full recovery

F—Female, M—Male, N/A—Not Available, SXT sulfamethoxazole-trimethoprim. * Antibiotic susceptibility testing was carried out using a variety of methods including disk diffusion testing, agar and broth dilution testing and E-testing methods.

## 6. Treatment of *Comamonas* spp. Infections

Antibiotic treatment of *Comamonas* spp. infections can be difficult. *Comamonas* spp. can be resistance to various antibiotic families including β-lactams (penicillins, cephalosporins and the development of resistance to carbapenems). To date, no controlled trials of antimicrobial therapies for *Comamonas* spp. infections in humans have taken place; consequently, antibiotic treatment ought to be based upon results of in vitro susceptibility testing on isolates. A variety of different antibiotics have been employed to treat *Comamonas* spp. infections found in the literature and, in most cases, they are susceptible to aminoglycosides, fluoroquinolones, carbapenems, piperacillin-tazobactam, trimethoprim-sulfamethoxazole, and cephalosporins (Table 2, Table 3 and Table 4).

Resistance to β-lactams class antimicrobials can be due to the possession of several genes by *Comamonas* spp. *C. testosteroni* S44 possesses a three-gene operon that codes for a Class A β-lactamases (resistance to benzylpenicillin, ampicillin, cefalexin, cefazolin, cefuroxime, ceftriaxone, and cefepime). These genes are *CzoA* (Class A β-lactamase encoding gene)—inhibits β-lactams antibiotics, *CzoR* (LysR type transcriptional regulator)—positively affects the expression of *CzoA*, and the *IscR* gene—enhances the regulatory effect of CzoR when bounded to its promoter region [106]. Several resistance genes were found in *C. kerstersii* 8943, including *tetA*, *strB*, *sul1*, *bla_OXA-_*_1_, *strA*, *sul2*, *catB3* and *floR*. The *bla_IMP–_*_8_ gene (giving resistance to β-lactam antibiotics) has been found in a *Comamonas thiooxydans* isolate, which caused a urinary tract infection. This isolate also had a novel class D beta-lactamase gene *bla*_OXA_ and a *aac(6′)-Ib-c gene* (resistance to aminoglycoside antibiotics). A variety of efflux pumps were also identified in the genomes of this bacterial isolate. [105]. A study in 2022 found another *Comamonas thiooxydans* isolate with a plasmid-based *bla_IMP–_*_1_ gene [107]. In a study by Hem et al., 2022, 32 *Comamonas. denitrificans* and 5 *C. testosteroni* from wastewater, 1 *C. denitrificans* from a wetland, and 1 *C. aquatica* from a lake with public access were sequenced. All were found to be resistant to carbapenem antibiotics. However, only 13 *C. denitrificans* isolates were found to have an identifiable carbapenemase *bla_GES_*_-5_. No identifiable carbapenemase genes were found in the other isolates. Other *C. denitrificans* isolates carried extended-spectrum b-lactamase (ESBL) *bla_OXA_* genes. This was the first report of resistance to carbapenem antibiotics in both *C. denitrificans* and *C. aquatica*; however, carbapenem-resistance was previously reported in a *C. testosteroni* infection in Turkey in 2015 [77,108].

## 7. Conclusions

*Comamonas* spp. are not currently considered important pathogens and are thought of as being of low virulence and of being a lesser danger in comparison to other non-fermenting Gram-negative bacteria such as *Pseudomonas aeruginosa*. Nevertheless, in this review, fifty-five separate outbreaks of *Comamonas* spp. infections have been identified from the scientific literature not taking into account unreported/undocumented cases. It must be recommended that the scientific community acknowledge the ability of this organism to elude antimicrobials and thus the potential for antimicrobial resistance transference between organisms, particularly in an era of growing antimicrobial susceptibility concerns.

## Figures and Tables

**Table 1:** Listing of validly published *Comamonas* species.

Species	Origin/Isolation Site	Genome Sequences	Reference
*Comamonas aquatica*	China/Freshwater River	Strain: CJG, Size: 3.76 Mb Ref Genome: GCA_000935165.2 (6 genomes)	Wauters et al., 2000 [28]
*Comamonas aquatilis*	Germany/Garden Pond	No Genome	Kampfer et al., 2018 [29]
*Comamonas badia*	Japan/Activated sludge	Strain: IAM 14839, Size: 3.68 Mb Ref Genome: GCA_000484635.1	Tago and Yokota, 2004 [30]
*Comamonas composti*	Taiwan/food waste compost	Strain: YY287T, Size: 4.63 Mb Ref Genome: GCA_000429845.1	(Young et al., 2008) [31]
*Comamonas denitrificans*	Sweden/Activated sludge	Strain: 123T Size: 3 Mb Ref Genome: GCA_017368815.1	Gumaelius et al., 2001 [32]
*Comamonas fluminis*	China/River water	Strain: CJ34T Size: 4.86 Mb Ref Genome: NZ_CP066783.1	Park et al., 2022 [33]
*Comamonas granuli*	Korea/Granules used in wastewater treatment plant	Strain: NBRC 101663T, Size: 3.51 Mb Ref Genome: GCA_003604195.1	Kim et al., 2008 [34]
*Comamonas guangdongensis*	China/Subterranean Forest sediment	No Genome	Zhang et al., 2013 [35]
*Comamonas humi*	Japan/Soil	No Genome	Hatayama, 2014 [36]
*Comamonas jiangduensis*	China/Agricultural soil	Strain: YW1T, Size: 2.76 Mb Ref Genome: GCA_902829245.1	Sun et al., 2013 [37]
*Comamonas kerstersii*	Dialysis effluent of a patient	Strain: 8943, Size: 3.55 Mb Ref Genome: GCA_002056725.1	Wauters et al., 2003 [28]
*Comamonas koreensis*	Korea/Wetland	Strain: YH12T, T50-37 Size: 5.3 Mb Ref Genome: GCA_014076495.1	Chang et al., 2002 [38]
*Comamonas nitrativorans*	Uruguay/Denitrifying reactor	Strain: 23310T, Size: 3.36 Mb Ref Genome: SAMN02746010	Etchebehere, 2001 [39]
*Comamonas odontotermitis*	Taiwan/Termite Odontotermes formosanus gut	Strain: Dant 3-8T, Size: 4.42 Mb. Ref Genome: GCA_020080045 (For WLL)	(Chou et al., 2007) [40]
*Comamonas phosphati*	China/Phosphate rock powder—from phosphate mine	Strain: WYH 22-41T, Size: 4.1 Mb Ref Genome: GCA_014637085.1	Fuhong et al., 2016 [41]
*Comamonas piscis*	Korea/Korean rockfish intestine	Strain: CN1T, Size: 5.2 Mb Ref Genome: GCA_014109725.1	Kang et al., 2016 [42]
*Comamonas sediminis*	USA/Lagoon sediments	Strain: S3T, Size: 4.42 Mb Ref Genome: JAFBFN010000000 (for 4487)	Subhash et al., 2016 [43]
*Comamonas serinivorans*	China/Wheat straw compost	Strain: SP-35T, Size: 4.52 Mb. Ref Genome: GCA_002158865.1	Daochen et al., 2014 [44]
*Comamonas suwonensis*	Republic of Korea/Stream water	Strain: EJ-4 Size: 4.72 Mb Ref Genome: GCA_012844455.2	Park et al. 2021 [45]
*Comamonas terrae*	Thailand/Agricultural soil	Strain: A3-3T, Size: 4.7Mb. Ref Genome: GCA_001544075.1	Chipirom et al., 2012 [46]
*Comamonas terrigena*	Boston/Hay infusion made from fresh water	Strain: NCIB 8193, Size: 4.7 Mb Ref Genome: AP019749.1	De Vos et al., 1985 [24]
*Comamonas testosteroni*	Organic compounds	Strain: KS 0043, Size: 5.41 Mb GCA_000241525.2 (21 Genomes)	Tamaoka et al., 1987 [47]
*Comamonas thiooxydans*	Sulphur spring	Strain: S23T, Size: 5.27 Mb Ref Genome: GCA_000964545.1	Pandey et al., 2009 [48]
*Comamonas zonglianii*	China/Phenol contaminated soil	No Genome	Xin-Yan et al., 2011 [49]

## Data Availability

Not applicable.

## References

[1] Ryan M.P., Adley C.C. (2014). *Ralstonia* spp.: Emerging global opportunistic pathogens. Eur. J. Clin. Microbiol. Infect. Dis..

[2] Ryan M.P., Pembroke J.T., Adley C.C. (2006). *Ralstonia pickettii*: A persistent Gram-negative nosocomial infectious organism. J. Hosp. Infect..

[3] Ryan M.P., Adley C.C. (2010). *Sphingomonas paucimobilis*: A persistent Gram-negative nosocomial infectious organism. J. Hosp. Infect..

[4] Coughlan A., Ryan M.P., Cummins N.M., Towler M.R. (2011). The response of *Pseudomonas aeruginosa* biofilm to the presence of a glass polyalkenoate cement formulated from a silver containing glass. J. Mater. Sci..

[5] Ryan M.P., Pembroke J.T. (2018). *Brevundimonas* spp: Emerging global opportunistic pathogens. Virulence.

[6] Ryan M.P., Pembroke J.T. (2020). The Genus *Ochrobactrum* as major opportunistic pathogens. Microorganisms.

[7] Abraham J.E.M., Simon G.L. (2007). *Comamonas testosteroni* bacteremia: A case report and review of the literature. Infect. Dis. Clin. Pract..

[8] Zhong F., Wu J., Dai Y., Yang L., Zhang Z., Cheng S., Zhang Q. (2015). Bacterial community analysis by PCR-DGGE and 454-pyrosequencing of horizontal subsurface flow constructed wetlands with front aeration. Appl. Microbiol. Biotechnol..

[9] Handschuh H., Ryan M.P., O’Dwyer J., Adley C.C. (2017). Assessment of the bacterial diversity of aircraft water: Identification of the frequent fliers. PLoS ONE.

[10] Xiong J., Li D., Li H., He M., Miller S.J., Yu L., Rensing C., Wang G. (2011). Genome analysis and characterization of zinc efflux systems of a highly zinc-resistant bacterium, *Comamonas testosteroni* S44. Res. Microbiol..

[11] Andrade G., Esteban E., Velasco L., Lorite M.J., Bedmar E.J. (1997). Isolation andiIdentification of N_2-_fixing microorganisms from the Rhizosphere of *Capparis spinosa* (L.). Plant Soil.

[12] Pavone S., Rinoldo R., Albini E., Fiorucci A., Caponi B., Fratto A., Manuali E., Papa P., Magistrali C.F. (2021). First report of urinary tract infection caused by *Comamonas kerstersii* in a goat. BMC Vet. Res..

[13] Wang Y.H., Huang Z., Liu S.J. (2019). Chemotaxis towards aromatic compounds: Insights from *Comamonas testosteroni*. Int. J. Mol. Sci..

[14] Boon N., Goris J., De Vos P., Verstraete W., Top E.M. (2000). Bioaugmentation of activated sludge by an indigenous 3-Chloroaniline- Degrading *Comamonas testosteroni* Strain, I2gfp. Appl. Environ. Microbiol..

[15] Liu L., Jiang C.Y., Liu X.Y., Wu J.F., Han J.G., Liu S.J. (2007). Plant-microbe association for rhizoremediation of chloronitroaromatic pollutants with *Comamonas* sp. strain CNB-1. Environ. Microbiol..

[16] Oppermann U.C.T., Belai I., Maser E. (1996). Antibiotic resistance and enhanced insecticide catabolism as consequences ofsSteroid induction in the gram-negative bacterium *Comamonas testosteroni. J. Steroid Biochem*. Mol. Biol..

[17] Wu J.F., Jiang C.Y., Wang B.J., Ma Y.F., Liu Z.P., Liu S.J. (2006). Novel partial reductive pathway for 4-Chloronitrobenzene and Nitrobenzene degradation in *Comamonas* sp. strain CNB-1. Appl. Environ. Microbiol..

[18] Shi Z., Qi X., Zeng X.A., Lu Y., Zhou J., Cui K., Zhang L. (2021). A newly isolated bacterium *Comamonas* Sp. XL8 alleviates the toxicity of cadmium exposure in rice seedlings by accumulating cadmium. J. Hazard. Mater..

[19] Staniland S., Coppock M., Tuffin M., van Zyl L., Roychoudhury A.N., Cowan D. (2010). Cobalt uptake and resistance to trace metals in *Comamonas testosteroni* isolated from a heavy-metal contaminated site in the Zambian copperbelt. Geomicrobiol. J..

[20] Farooq S., Farooq R., Nahvi N. (2017). *Comamonas testosteroni*: Is it still a rare human pathogen. Case Rep. Gastroenterol..

[21] Kim H.J., Lee Y., Oh K., Choi S.-H., Sung H., Huh J.W. (2015). Septic shock due to unusual pathogens, *Comamonas testosteroni* and *Acinetobacter guillouiae* in an immune competent patient. Korean J. Crit. Care Med..

[22] Cooper G.R., Staples E.D., Iczkowski K.A., Clancy C.J. (2005). *Comamonas* (*Pseudomonas*) *testosteroni* endocarditis. Cardiovasc. Pathol..

[23] Davis G.H., Park R.W. (1962). A taxonomic study of certain bacteria currently classified as *Vibrio* species. J. Gen. Microbiol..

[24] De Vos P., Kersters K., Falsen E. (1985). *Comamonas* Davis and Park 1962 Gen. Nov., Nom. Rev. Emend., and *Comamonas terrigena* Hugh 1962 Sp. Nov., Nom. Rev. Int. J. Syst. Bacteriol..

[25] Wen A., Fegan M., Hayward C., Chakraborty S., Sly L.I. (1999). Phylogenetic Relationships among Members of the *Comamonadaceae*, and Description of *Delftia acidovorans* (Den Dooren de Jong 1926 and Tamaoka et Al. 1987) Gen. Nov., Comb. Nov. Int. J. Syst. Bacteriol..

[26] Tamura K., Stecher G., Kumar S. (2021). MEGA11: Molecular Evolutionary Genetics Analysis Version 11. Mol. Biol. Evol..

[27] Ryan M.P., Adley C.C., Pembroke J.T., Méndez-Vilas A. (2013). The use of MEGA as an educational tool for examining the phylogeny of antibiotic resistance genes. Microbial Pathogens and Strategies for Combating Them: Science, Technology and Education.

[28] Wauters G., De Baere T., Willems A., Falsen E., Vaneechoutte M. (2003). Description of *Comamonas aquatica* Comb. Nov. and *Comamonas kerstersii* sp. nov. for two subgroups of *Comamonas terrigena* and emended description of *Comamonas terrigena*. Int. J. Syst. Evol. Microbiol..

[29] Kämpfer P., Busse H.J., Baars S., Wilharm G., Glaeser S.P. (2018). *Comamonas aquatilis* sp. Nov., isolated from a garden pond. Int. J. Syst. Evol. Microbiol..

[30] Tago Y., Yokota A. (2004). *Comamonas badia* sp. nov., A floc-forming bacterium isolated from Activated Sludge. J. Gen. Appl. Microbiol..

[31] Young C.C., Chou J.H., Arun A.B., Yen W.S., Sheu S.Y., Shen F.T., Lai W.A., Rekha P.D., Chen W.M. (2008). *Comamonas composti* sp. nov., isolated from food waste compost. Int. J. Syst. Evol. Microbiol..

[32] Gumaelius L., Magnusson G., Pettersson B., Dalhammar G. (2001). *Comamonas denitrificans* sp. nov., an efficient denitrifying bacterium isolated from Activated Sludge. Int. J. Syst. Evol. Microbiol..

[33] Park E.H., Kim Y.S., Cha C.J. (2022). *Comamonas fluminis* sp. nov., isolated from the Han River Republic of Korea. Int. J. Syst. Evol. Microbiol..

[34] Kim K.H., Ten L.N., Liu Q.M., Im W.T., Lee S.T. (2008). *Comamonas granuli* sp. nov., isolated from granules used in a Wastewater Treatment Plant. J. Microbiol..

[35] Zhang J., Wang Y., Zhou S., Wu C., He J., Li F. (2013). *Comamonas guangdongensis* sp. nov., isolated from subterranean forest sediment, and emended description of the Genus *Comamonas*. Int. J. Syst. Evol. Microbiol..

[36] Hatayama K. (2014). *Comamonas humi* sp. nov., Isolated from Soil. Int. J. Syst. Evol. Microbiol..

[37] Sun L.N., Zhang J., Chen Q., He J., Li Q.F., Li S.P. (2013). *Comamonas jiangduensis* Sp. Nov., a biosurfactant producing bacterium isolated from agricultural soil. Int. J. Syst. Evol. Microbiol..

[38] Chang Y.H., Han J.-I., Chun J., Lee K.C., Rhee M.S., Kim Y.B., Bae K.S. (2002). *Comamonas koreensis* sp. nov., a non-motile species from wetland in Woopo, Korea. Int. J. Syst. Evol. Microbiol..

[39] Etchebehere C., Errazquin M.I., Dabert P., Moletta R., Muxí L. (2001). *Comamonas nitrativorans* sp. nov., a novel denitrifier isolated from a denitrifying reactor treating landfill leachate. Int. J. Syst. Evol. Microbiol..

[40] Chou J.H., Sheu S.Y., Lin K.Y., Chen W.M., Arun A.B., Young C.C. (2007). *Comamonas odontotermitis* sp. nov., isolated from the gut of the termite *Odontotermes formosanus*. Int. J. Syst. Evol. Microbiol..

[41] Xie F., Ma H., Quan S., Liu D., Chen G. (2016). *Comamonas phosphati* Nov., sp. nov., isolated from a phosphate mine. Int. J. Syst. Evol. Microbiol..

[42] Kang W., Kim P.S., Hyun D.W., Lee J.Y., Kim H.S., Oh S.J., Shin N.R., Bae J.W. (2016). *Comamonas piscis* sp. nov., isolated from the intestine of a Korean rockfish, *Sebastes schlegelii*. Int. J. Syst. Evol. Microbiol..

[43] Subhash Y., Bang J.J., You T.H., Lee S.S. (2016). Description of *Comamonas sediminis* sp. nov., isolated from lagoon sediments. Int. J. Syst. Evol. Microbiol..

[44] Zhu D., Xie C., Huang Y., Sun J., Zhang W. (2014). Description of *Comamonas serinivorans* sp. nov., isolated from wheat straw compost. Int. J. Syst. Evol. Microbiol..

[45] Park K.H., Yu Z., Dong K., Lee S.S. (2021). *Comamonas suwonensis* sp. nov., isolated from stream water in the Republic of Korea. Int. J. Syst. Evol. Microbiol..

[46] Chitpirom K., Tanasupawat S., Akaracharanya A., Leepepatpiboon N., Prange A., Kim K.W., Lee K.C., Lee J.S. (2012). *Comamonas terrae* sp. nov., an arsenite-oxidizing bacterium isolated from agricultural soil in Thailand. J. Gen. Appl. Microbiol..

[47] Tamaoka J., Ha D.M., Komagata K. (1987). Reclassification of *Pseudomonas acidovorans* Den Dooren de Jong 1926 and *Pseudomonas testosteroni* Marcus and Talalay 1956 as *Comamonas acidovorans* comb. nov. and *Comamonas testosteroni* comb. nov., with an Emended Description of the Genus *Comamonas*. Int. J. Syst. Bacteriol..

[48] Narayan K.D., Pandey S.K., Das S.K. (2010). Characterization of *Comamonas thiooxidans* sp. nov., and comparison of thiosulfate oxidation with *Comamonas testosteroni* and *Comamonas composti*. Curr. Microbiol..

[49] Yu X.Y., Li Y.F., Zheng J.W., Li Y., Li L., He J., Li S.P. (2011). *Comamonas zonglianii* sp. nov., isolated from phenol contaminated soil. Int. J. Syst. Evol. Microbiol..

[50] Willems A., Gillis M. (2015). Comamonas. Bergey’s Manual of Systematics of Archaea and Bacteria.

[51] Willems A., De Vos P., Dworkin M., Falkow S., Rosenberg E., Schleifer K.-H., Stackebrandt E. (2006). Comamonas. The Prokaryotes.

[52] Public Health England (2015). UK Standards for Microbiology Investigations Identification of *Pseudomonas* species and other Non-Glucose Fermenters. Issued by the Standards Unit, Public Health England. https://assets.publishing.service.gov.uk/government/uploads/system/uploads/attachment_data/file/422699/ID_17i3.pdf.

[53] Wu Y., Zaiden N., Cao B. (2018). The Core- and Pan-Genomic Analyses of the Genus *Comamonas*: From environmental adaptation to potential virulence. Front. Microbiol..

[54] Barbaro D.J., Mackowiak P.A., Barth S.S., Southern P.M. (1987). *Pseudomonas testosteroni* Infections: Eighteen Recent Cases and a Review of the Literature. Rev. Infect. Dis..

[55] Jin L., Perper J.A., Cina S.J. (2008). *Comamonas testosteroni* meningitis in a homeless man. J. Forensic Sci..

[56] Swain B., Rout S. (2015). *Comamonas testosteroni* bacteraemia in a Tertiary Care Hospital. Indian J. Med. Microbiol..

[57] Yasayancan N., Koseoglu H.I. (2017). The 20th *Comamonas testosteroni* bacteremia case in the Literature from Turkey: Mortal and polymicrobial a case report and literature review. Eurasian J. Med. Oncol..

[58] Çetin Ş., Baslarli S., Celik B., Celik I. (2018). Pneumonia case by caused *Comamonas testosteroni* in pediatric intensive care unit. Eurasian J. Med. Oncol..

[59] Atkinson B.E., Smith D.L., Lockwood W.R. (1975). *Pseudomonas testosteroni* septicemia. Ann. Intern. Med..

[60] Smith E.G. (1979). *Pseudomonas testosteroni* pyarthrosis and septicemia. Clin. Microbiol. Newsl..

[61] Franzetti F., Cernuschi M., Esposito R., Moroni M. (1992). *Pseudomonas* Infections in Patients with AIDS and AIDS-related Complex. J. Intern. Med..

[62] Le Moal G., Paccalin M., Breux J.P., Roblot F., Roblot P., Becq-Giraudon B. (2001). Central venous catheter-related infection due to *Comamonas testosteroni* in a woman with breast cancer. Scand. J. Infect. Dis..

[63] Arda B., Aydemir S., Yamazhan T., Hassan A., Tünger A., Serter D. (2003). *Comamonas testosteroni* meningitis in a patient with recurrent cholesteatoma. APMIS.

[64] Smith M.D., Gradon J.D. (2003). Bacteremia due to *Comamonas* species possibly associated with exposure to tropical fish. South. Med. J..

[65] Gul M., Ciragil P., Bulbuloglu E., Aral M., Alkis S., Ezberci F. (2007). *Comamonas testosteroni* bacteremia in a patient with perforated acute appendicitis. Acta Microbiol. Immunol. Hung..

[66] Carolo G., Ganau M., De Micheli E., Gerosa M., Solbiati M. (2007). P1466 *Comamonas testosteroni* Spondylodiscitis. Int. J. Antimicrob. Agents.

[67] Reddy A.K., Murthy S.I., Jalali S., Gopinathan U. (2009). Post-Operative endophthalmitis due to an unusual pathogen, *Comamonas testosteroni*. J. Med. Microbiol..

[68] Katircioǧlu K., Özkalkanli M.Y., Yurtsever S.G., Savaci S. (2010). Yoǧun Bakim Hastasinda *Comamonas testosteroni* Enfeksiyonu. Turk Anesteziyoloji Reanimasyon Dern. Derg..

[69] Nseir W., Khateeb J., Awawdeh M., Ghali M. (2011). Catheter-related bacteremia caused by *Comamonas testosteroni* in a hemodialysis patient. Hemodial. Int..

[70] Ozden S., Kocturk S.A., Guler S., Kilinc D. (2011). Comamonas testoster Noni Infection in Intensive Care Patient.

[71] Tsui T.L., Tsao S.M., Liu K., Chen T.Y., Wang Y.L., Teng Y.H., Lee Y.T. (2011). Comamonas testosteroni infection in Taiwan: Reported Two cases and literature review. J. Microbiol. Immunol. Infect..

[72] Farshad S., Norouzi F., Aminshahidi M., Heidari B., Alborzi A. (2012). Two Cases of Bacteremia Due to an Unusual Pathogen, *Comamonas testosteroni* in Iran and a Review Literature. J. Infect. Dev. Ctries..

[73] Al Ramahi J.W., Rumoh S.A., Khali B.W. (2013). *Comamonas testosteroni* Blood Stream Infection in a patient with end-stage renal failure on hemodialysis. Int. Arab. J. Antimicrob. Agents.

[74] Bayhan G.I., Tanir G., Karaman I., Özkan Ş. (2013). *Comamonas testosteroni*: An unusual bacteria associated with acute appendicitis. Balkan Med. J..

[75] Altun E., Kaya B., Taktakoǧlu O., Karaer R., Paydas S., Balal M., Seyrek N. (2013). *Comamonas testosteroni* peritonitis secondary to dislocated intrauterine device and laparoscopic intervention in a continuous ambulatory peritoneal dialysis patient. Perit. Dial. Int..

[76] Orsini J., Tam E., Hauser N., Rajayer S. (2014). Polymicrobial bacteremia involving *Comamonas testosteroni*. Case Rep. Med..

[77] Duran A., Okur F., Sahin V., Uyar I., Abacilar A., Akpinar M., Alayunt E., Ates M. (2015). *Comamonas testosteroni* endocarditis in Turkey: A case report and review of the literature. Int. Med. J. Sifa Univ..

[78] Khalki H., Deham H., Taghouti A., Yahyaoui G., Mahmoud M. (2016). Appendicite à *Comamonas testosteroni*. Med. Mal. Infect..

[79] Pekintürk N., Akgüneş A. (2016). Nadir Bir Patojen *Comamonas testosteronı*: Olgu Sunumu Ve Literatürün Gözden Geçirilmesi. Kocaeli Üniversitesi Sağlık Bilim. Derg..

[80] Parolin M., Baraldi M., Valentini E., Murer L., Vidal E. (2016). *Comamonas testosteroni*-associated peritonitis in a pediatric peritoneal dialysis patient. World J. Nephrol..

[81] Hung Y.M., Chang Y.T., Kao C.H. (2017). Polymicrobial Bacteremia Involving *Comamonas testosteroni* in a Patient on Dialysis with Acute Appendicitis. Ther. Apher. Dial..

[82] Ruziaki W., Hashami H. (2017). Unusual pathogen *Comamonas testosteroni* sepsis following gastroenteritis in a 12 months old child: Case report and literature review. Am. J. Med. Case Reports.

[83] Tartar A.S., Tartar T. (2020). A rare pathogen in acute appendicitis: Two cases with *Comamonas testosteroni* infection and literature review. J. Pediatr. Infect. Dis..

[84] Lovell A.R.O., Forde C.A. (2019). *Comamonas testosteroni* bacteremia in a young male with mancreatitis: A case peport. J. Clin. Case Rep..

[85] Tiwari S., Nanda M. (2019). Bacteremia caused by *Comamonas testosteroni* an unusual pathogen. J. Lab. Physicians.

[86] Buyukberber S.G., Mumcuoglu I., Ozbay B.O., Aypak A., Dinc B. A Rare Pathogen *Comamonas Testosteroni*: A Case Report and Review of the Literature, 14 July 2021, Preprint (Version 1).

[87] Miloudi M., El Kamouni Y., Oulhadj H., Arsalane L., Zouhair S. (2021). *Comamonas testosteroni* appendicitis: About a case and review of the Llterature. Infect. Dis. Now.

[88] Ayhanci T., Demiray T., Özmen E., İnce B., Sadeq M., Aydin A., Yaylaci S. (2021). A rare case of bacteriemia due to *Comamonas testosteroni*. J. Biotechnol. Strateg. Heal. Res..

[89] Sammoni A., Abdalah A., Al-Aissami M. (2022). *Comamonas testosteroni* bacteremia: A rare unusual pathogen detected in a burned patient: Case report and literature review. Ann. Med. Surg..

[90] Almuzara M.N., Cittadini R., Ocampo C.V., Bakai R., Traglia G., Ramirez M.S., Del Castillo M., Vay C.A. (2013). Intra-abdominal infections due to *Comamonas kerstersii*. J. Clin. Microbiol..

[91] Biswas J.S., Fitchett J., O’Hara G. (2014). *Comamonas kerstersii* and the Perforated Appendix. J. Clin. Microbiol..

[92] Opota O., Ney B., Zanetti G., Jaton K., Greub G., Prod’hom G. (2014). Bacteremia caused by *Comamonas kerstersii* in a Patient with diverticulosis. J. Clin. Microbiol..

[93] Almuzara M., Barberis C., Veiga F., Bakai R., Cittadini R., Vera Ocampo C., Alonso Serena M., Cohen E., Ramirez M.S., Famiglietti A. (2017). Unusual presentations of *Comamonas kerstersii* infection. New Microbes New Infect..

[94] Almuzara M., Cittadini R., Estraviz M.L., Ellis A., Vay C. (2018). First Report of *Comamonas kerstersii* causing urinary tract infection. New Microbes New Infect..

[95] Zhou Y.H., Ma H.X., Dong Z.Y., Shen M.H. (2018). *Comamonas kerstersii* bacteremia in a patient with acute perforated appendicitis. Medicine.

[96] Liu X.J., Qiao X.W., Huang T.M., Li L., Jiang S.P. (2020). *Comamonas kerstersii* Bacteremia. Med. Mal. Infect..

[97] Palacio R., Cabezas L., Cornejo C., Seija V. (2020). *Comamonas kerstersii* bacteremia in a young man with acute appendicitis. Rev. Chil. Infectol..

[98] Farfán-Cano G., Parra-Vera H., Ávila-Choez A., Silva-Rojas G., Farfán-Cano S. (2020). First identification in Ecuador of *Comamonas kerstersii* as an infectious agent. Rev. Chil. Infectol..

[99] Farfán-Cano G.G., Sarmiento-Bobadilla J.A., León E.A.J., Crespo-Díaz C.M., Silva-Rojas G.A., Parra-Vera H.J., Solórzano-Bravo M.T., Chantong-Villacres L.A., Silva-Rojas K.J. (2021). *Comamonas kerstersii* Strains on in patients with acute Appendicitis: Review of literature and case report. Interam. J. Med. Heal..

[100] Rong K., Delport J., AlMutawa F. (2022). *Comamonas kerstersii* bacteremia of unknown origin. Case Rep. Infect. Dis..

[101] Bennani H., El Ouarradi A., Hanchi A.L., Soraa N. (2022). A young child with acute perforated appendicitis due to Comamonas kerstersii: A rare case report. Pan Afr. Med. J..

[102] Sonnenwirth A.C. (1970). Bacteremia with and without meningitis due to *Yersinia enterocolitica*, *Edwardsiella tarda, Comamonas terrigena*, and *Pseudomonas maltophilia*. Ann. N. Y. Acad. Sci..

[103] Isotalo P.A., Edgar D., Toye B. (2000). Polymicrobial tenosynovitis with *Pasteurella multocida* and other gram-negative Bacilli after a Siberian Tiger bite. J. Clin. Pathol..

[104] Kaeuffer C., Schramm F., Meyer A., Hansmann Y., Guffroy A., Argemi X. (2018). First case of *Comamonas aquatica* bacteremia complicated by septic shock. Médecine Mal. Infect..

[105] Guo X., Wang Q., Xu H., He X., Guo L., Liu S., Wen P., Gou J. (2021). Emergence of IMP-8-Producing *Comamonas thiooxydans* causing Urinary Tract Infection in China. Front. Microbiol..

[106] Zhuang W., Liu H., Li J., Chen L., Wang G. (2017). Regulation of Class A β-Lactamase *czoA* by *czoR* and *iscR* in *Comamonas testosteroni* S44. Front. Microbiol..

[107] Suzuki Y., Nakano R., Nakano A., Tasaki H., Asada T., Horiuchi S., Saito K., Watanabe M., Nomura Y., Kitagawa D. (2022). *Comamonas thiooxydans* expressing a plasmid encoded IMP-1 Carbapenemase isolated from continuous ambulatory peritoneal dialysis of an inpatient in Japan. Front. Microbiol..

[108] Hem S., Wyrsch E.R., Drigo B., Baker D.J., Charles I.G., Donner E., Jarocki V.M., Djordjevic S.P. (2022). Genomic Analysis of Carbapenem-Resistant *Comamonas* in Water Matrices: Implications for Public Health and Wastewater Treatments. Appl. Environ. Microbiol..

